# Effect of Ketamine on the Bispectral Index, Spectral Edge Frequency, and Surgical Pleth Index During Propofol-Remifentanil Anesthesia: An Observational Prospective Trial

**DOI:** 10.1213/ANE.0000000000007255

**Published:** 2024-11-01

**Authors:** Federico Linassi, Carla Troyas, Matthias Kreuzer, Leonardo Spanò, Paolo Burelli, Gerhard Schneider, Paolo Zanatta, Michele Carron

**Affiliations:** From the * Department of Pharmaceutical and Pharmacological Sciences, Università degli Studi di Padova, Padova, Italy; † Department of Anesthesiology and Critical Care, Treviso Regional Hospital, AULSS 2 Marca Trevigiana Piazzale Ospedale, Treviso, Italy; ‡ Department of Anesthesiology and Intensive Care, Klinikum rechts der Isar, Technical University of Munich, School of Medicine and Health, München, Germany; § Department of Medicine—DIMED, Section of Anesthesiology and Intensive Care, University of Padova, Padova, Italy.

## Abstract

**BACKGROUND:**

Ketamine administration during stable propofol anesthesia is known to be associated with an increase in bispectral index (BIS) but a “deepening” in the level of hypnosis. This study aimed to evaluate the association between the effect-site concentration of ketamine (CeK) and 2 electroencephalogram (EEG)-derived parameters, the BIS and spectral edge frequency (SEF_95_), after the administration of a ketamine bolus. Secondary aims included investigating the BIS and SEF_95_ variations with time and changes in the surgical pleth index (SPI).

**METHODS:**

We conducted an observational, prospective, single-center study analyzing intraoperative data from 14 adult female patients undergoing breast oncologic surgery. During stable propofol-remifentanil target-controlled infusion (TCI) anesthesia, a ketamine analgesic bolus was delivered with the target CeK set to 1 μg.mL^−1^ (Domino model) corresponding to a dose of 0.57 mg.kg^−1^ (interquartile range [IQR] 0.56–0.57 mg.kg^−1^). Once the CeK reached a value of 1 μg.mL^−1^, the target CeK was set to 0 μg.mL^−1^. We determined the median BIS, SEF_95_, and SPI trends with time and as a function of the modeled CeK.

**RESULTS:**

BIS and SEF_95_ showed no significant change from when ketamine was administered to when CeK=1 μg.mL^−1^, but a significant increase was observed at lower CeKs. The maximum BIS was reached at 16.0 minutes [10.2–22.7 minutes] after CeK=1 μg.mL^−1^, at CeK=0.22 μg.mL^−1^ [0.12–0.41 μg.mL^−1^]. The peak SEF_95_ value was observed at 10.0 minutes [8.62–14.1 minutes] after CeK=1 μg.mL^−1^, at CeK=0.43 μg.mL^−1^ [0.25–0.50 μg.mL^−1^]. No significant association was found between CeK and the registered SPI values.

**CONCLUSIONS:**

Our results show that BIS and SEF_95_, but not SPI, follow a CeK-dependent trend after administering a ketamine bolus. Interestingly, their peak values were not reached at CeK=1 μg.mL^−1^, but after several minutes after the drug infusion at CeKs in the 0.2 to 0.5 μg.mL^−1^ range. This may be explained by the specific pharmacodynamics of ketamine and its varying effects at different concentrations, as well as by the time delay associated with the calculation of the BIS.

**KEY POINTS**

**Question**: How does ketamine infusion during propofol-remifentanil anesthesia influence the bispectral index (BIS), spectral edge frequency (SEF_95_), and surgical pleth index (SPI)?

**Findings:** BIS and SEF_95_, but not SPI, showed a biphasic trend concerning the effect-site concentration of ketamine after administering a ketamine bolus. The BIS and SEF_95_ peak values were reached several minutes after the infusion of the drug.

**Meaning**: The anesthesiologist should know ketamine’s influence on these indexes to optimally titrate anesthesia.

**See Article, page 1273**

The perioperative use of ketamine, a noncompetitive antagonist of the ionotropic *N*-methyl-D-aspartic acid (NMDA) glutamate receptor, can reduce postoperative pain intensity and consumption of analgesic drugs.^1^ Ketamine is often used in the context of multimodal general anesthesia. This pharmacologic approach combines analgesics, adjuvants, and local anesthetics for perioperative nociception management, aiming to improve pain relief while reducing opioid administration and opioid-related adverse effects.^2^ For this reason, ketamine at subanesthetic concentrations has been suggested to be used in the perioperative care of patients, including during breast surgery, where postoperative pain, depression, and mood disorders may complicate the postoperative course.^3^ For these patients, ketamine has been indicated to be a safe and effective drug with significant analgesic and antidepressant properties that do not increase the risk of adverse events. The effect of ketamine on the electroencephalogram (EEG) has been described previously in the literature. When ketamine was administered alone in subanesthetic concentrations, the EEG developed fast oscillations in the high-beta and low-gamma range (25–32 Hz).^4^ When administered during propofol anesthesia with its typical patterns of delta-dominant and alpha-dominant oscillations,^4^ ketamine shifted the spectral α-peak to higher frequencies by approximately 5 Hz.^5^ In addition, further studies have shown that, while a bolus of ketamine (0.5 mg.kg^−1^) increased the bispectral index (BIS)—an EEG-derived parameter for monitoring the hypnotic effects of anesthetics—a smaller ketamine bolus of 0.2 mg.kg^−1^ did not.^6,7^ A dose-dependent effect of ketamine has been suggested,^6^ but data supporting this assumption are not available. Total Intravenous Anesthesia with Target Controlled Infusion (TIVA-TCI) regimes could help evaluate a dose-dependent effect.

TCI utilizes pharmacokinetic drug models to deliver hypnotic and analgesic drugs to achieve stable estimated plasma and effect-site concentration of the hypnotic drug, such as propofol, and the analgesic drug, such as remifentanil.^8^ The dose delivered by a TCI system depends on the pharmacokinetic-pharmacodynamic model used, patient characteristics, and target concentration. Since the drug’s effect is more closely related to blood concentration than infusion rate, drug delivery via TCI can lead to stable blood concentrations of intravenous anesthetics and analgesics. It may help to better understand their dose-dependent effect.^8^ Among various TCI models for ketamine, the Domino model has been used for low-dose TCI administration of ketamine in experimental settings.^9,10^

The primary aim of this study was to evaluate the dose-dependent effect of ketamine on 2 EEG-derived parameters, the BIS and spectral edge frequency (SEF_95_), as well as on an index for nociception monitoring, the surgical pleth index (SPI), after the administration of an analgesic ketamine bolus during stable TIVA-TCI.

These parameters are often used to monitor anesthesia’s hypnotic and analgesic levels. Thus, a better understanding of how ketamine affects these parameters can help anesthesiologists to better guide drug administration based on these parameters.

## METHODS

### Study Design

This study was approved by the Ethical Committee of Treviso Regional Hospital, Italy (N. 681/CE Marca), and written informed consent was obtained from all subjects participating in the trial. The trial was registered before patient enrollment at clinicaltrials.gov (NCT05288764, principal investigator: F. Linassi, date of registration: March 10, 2022).

This article adheres to the applicable Equator guidelines. The corresponding Strengthening the Reporting of Observational Studies in Epidemiology (STROBE) flow diagram is available as Supplemental Digital Content 1, Figure S1, http://links.lww.com/AA/F49.

### Study Population

Adult female patients scheduled for breast surgery (mastectomy) from March 31, 2022 to January 15, 2023 at the Treviso Regional Hospital with a planned surgery duration of >90 minutes under TIVA-TCI anesthesia were recruited. Exclusion criteria included an American Society of Anesthesiologists (ASA) physical status >II, administration of neuromuscular blocking agents, neurological, cerebrovascular, or psychiatric disease, as well as the suffering from severe respiratory, cardiovascular, kidney, or liver disease. In addition, patients in continuous therapy with antidepressant drugs or anxiolytics or with a history of alcohol, drug, or psychoactive drug abuse, as well as those requiring perioperative anxiolysis or intraoperative vasoactive drugs, were excluded from this study.

### General Anesthesia

After overnight fasting and before anesthesia induction, standard vital signs monitoring, including a continuous electrocardiogram, pulse oximetry, and a noninvasive blood pressure measurement, were applied. An intravenous line was placed in the arm in preparation for TIVA-TCI. A BIS bilateral sensor (Medtronic, Dublin, Ireland) was placed on the patient’s forehead to capture the EEG signal and was connected to an XP monitor to monitor the BIS (Monitor BIS Module Vista Revision 3.50). BIS values between 40 and 60 seem adequate for general anesthesia during surgery. The BIS monitor also provides a value for the SEF_95_, the frequency below which 95% of the power within a defined frequency range lies. Previous studies reported an adequate anesthetic level at SEF_95_ = 8 to 13 Hz.^11^ SEF_95_ trend data were recorded for all patients. The SPI was used to monitor the hemodynamic response to surgical stimuli. The SPI is a normalized score based on the photoplethysmographic analysis of the pulse wave and the heartbeat interval, used for nociception monitoring during general anesthesia. SPI target values between 20 and 50 are recommended during general anesthesia.^12^

Induction and maintenance of anesthesia were performed using a TIVA-TCI delivery system. The target concentrations at the effect-site of propofol (CeP) and remifentanil (CeR) were achieved using the uSP6000 syringe pump infusion system (Arcomed AG) according to the Eleveld^13^ and Minto^14^ pharmacokinetic models. The initial CeP was set to 2 to 3 μg.mL^−1^ (for patients >50 years of age) or 3 to 4 μg.mL^−1^ (<50 years).^8^

Remifentanil TCI was started immediately after the loss of responsiveness (LoR), before the LMA placement, with initial CeR set to 2 ng.mL^−1^ (for patients >50 years of age) or 3 ng.mL^−1^ (<50 years).^8^ After remifentanil reached the initial target concentration, a laryngeal mask was placed to allow lung ventilation (Primus Anesthesia Workstation, Draeger). Propofol and remifentanil adjustments for targeting a BIS of 40 to 60 and an SPI of 20 to 50^12^ were made after induction during the first maintenance phase when surgery was started. Stable CeP and CeR were achieved by adjusting the CeP and CeR in 0.5 μg.mL^−1^ steps at intervals of ≥1 minute until BIS and SPI reached the suggested range. CeP and CeR were maintained for at least 5 one-minute intervals to confirm their steady state.^15^ CeP and CeR were not further adjusted during maintenance or after the ketamine bolus with the after effect-site concentration of ketamine (CeK) decrementing phase.

Ketamine was delivered using the uSP6000 syringe pump infusion system (Arcomed AG) after the Domino pharmacokinetic model. CeK was initially targeted to 1 μg.mL^−1^, corresponding to a recommended ketamine antalgic dose.^1–6^ Once the CeK reached 1 μg.mL^−1^, it was targeted to 0 μg.mL^−1^. Patients who required CeP or CeR adjustments during the CeK registration until it decreased to ≤0.05 μg.mL^−1^ were excluded from the study. At the end of the surgery, the TIVA-TCI system was readjusted to a target CeP of 0 μg.mL^−1^ and a CeR of 0 ng.mL^−1^.

### Variables and Clinical End Points

Variables recorded included age (years), body mass index (BMI, kg.m^−2^), ASA physical status classification, CeP (μg.mL^−1^), CeR (ng ml^−1^), CeK (μg.mL^−1^), duration of propofol infusion (min), total propofol dose and ketamine dose administered (total mg; mg.kg^−1^), EEG indexes and derived parameters (BIS, SEF_95_, burst suppression ratio [BSR]), and the SPI. The BIS and SEF_95_ were recorded by the BIS monitor with a per-second resolution. The CeK and SPI values were manually collected every minute starting 5 minutes before ketamine administration (baseline condition) until 60 minutes after CeK reached 1 µg.mL^−1^. The baseline recording period began when all the following criteria were met: stable CeP and CeR for at least 10 minutes, stable in-range EEG indices and SPI, stable respiratory and hemodynamic parameters, and first stages of surgery dissection completed.^16^

### EEG Recording and Preprocessing

The EEG traces were recorded with a sampling frequency of 128 Hz from the 4 frontal BIS sensors, live exported to a USB stick, and stored as.r4a files. The EEG traces from the.r4a files were extracted using custom MATLAB R2023a scripts (The Mathworks Inc). The recorded EEG signals were band-pass filtered from 0.5 to 40 Hz using a Butterworth forward-backward zero-phase band-pass routine using MATLAB’s *filtfilt* function. All EEG samples above the 100 µV threshold were considered artifacts and excluded from further EEG analysis.

### Processed EEG

Welch’s method with a uniform fast Fourier transform (NFFT) of 512 was used to calculate the power spectral density for EEG episodes of 5 seconds. The density spectral array (DSA) was constructed using a shift window of 1 second.

### Processed EEG Indexes (BIS and SEF_95_) Analysis

The monitoring system’s USB export was also used to record the BIS and SEF_95_ trend data stored in a.spa text file. The.spa file contained the processed EEG indices derived from the right and left temple electrodes with a per-second resolution. We synched the processed indices with the CeK data (1 value per minute) by interpolating the CeK trends using MATLAB’s function *interp1* to yield a CeK value for every second. To evaluate the effect of the initial ketamine dose, we tracked the change in SEF_95_ and BIS from the denoted time of delivery until CeK reached 1 μg.mL^−1^. We defined this period as the rapid administration phase. For all subjects, the time interval from the ketamine administration to when CeK=1 μg.mL^−1^ did not exceed 2 minutes. After reaching a CeK of 1 μg.mL^−1^, we aligned the BIS and SEF_95_ with the decreasing CeK. We calculated the mean BIS and SEF_95_ for every patient at every CeK from 1 to 0.04 μg.mL^−1^ in decreasing intervals of 0.05 ± 0.025 μg.mL^−1^. The base values for BIS and SEF_95_ were calculated for each patient as the mean over a 10-minute interval preceding the ketamine administration (11 minutes to 1 minute before the ketamine bolus). In addition, we plotted the group median (± median absolute deviation) BIS and SEF_95_ time trends for the time interval 10 minutes before to 55 minutes after the administration of ketamine.

### SPI Analysis

To explore changes in SPI as a function of CeK, we calculated the SPI values relative to its initial value at CeK = 1 μg.mL^−1^ in the range CeK = 1 μg.mL^−1^ to CeK = 0.04 μg.mL^−1^ in decreasing intervals of 0. 2 ± 0.1 μg.mL^−1^.

### Statistical Analysis

The sample size was based on the following assumptions: the increase between mean baseline BIS and maximum BIS after a ketamine bolus of dose 0.5 mg·kg^-1^ during propofol^7^ and sevoflurane anesthesia,^17^ was reported to be 23.3 and 13, respectively; type I error equal to 0.05; type II error equal to 0.05 (power [1^−^β] = 0.95); missing data assumed in about 50% of the patients. Considering these assumptions, the sample size was calculated as 14 patients. All numerical results are reported as median [interquartile range (IQR)].

#### BIS, SEF_95_, and SPI Analysis

To determine if the BIS and SEF_95_ changed significantly with CeK after the administration of ketamine, we performed Friedman’s test on the values of these parameters at baseline and every CeK from 1 to 0.05 μg.mL^−1^ in decreasing intervals of 0. 05 ± 0.025 μg.mL^−1^. The same approach was used to test for significant differences in the SPI value at baseline and at every CeK from 1 to 0.05 μg.mL^−1^ in decreasing intervals of 0. 2 ± 0.1 μg.mL^−1^. In addition, the statistical significance of BIS and SEF_95_ at each time point after ketamine administration compared to their baseline value was determined by applying the pairwise Wilcoxon signed-rank test. To correct for multiple comparisons, we only considered results significant if they occurred in neighboring time points. This neighbor-based approach was used in previous publications for EEG power spectral densities and trend data.^18^ We did not use the classical post hoc testing as the consequence of false negatives may be more costly than the erroneous report of false positives.^19^ Instead, we used the neighbor approach. For all statistical tests, a value of *P* < .05 was regarded as significant.

#### Model Fitting

Using the least squares method, we generated models for BIS and SEF95 as a function of CeK to fit a polynomial model of order 2. For each index, we generated the regression curve and determined the R^2^ value as a measure of the strength of the correlation.

## RESULTS

Of the 63 female patients screened for eligibility, 19 were excluded for not meeting inclusion criteria, 20 for not reaching CeK ≤0.05 during a stable CeP and CeR anesthesia, 9 declined to participate, and 1 was not included in the data analysis due to incomplete data collection (Supplemental Digital Content 1, Figure S1, http://links.lww.com/AA/F49). A total of 14 patients were included in the final analysis. No patient experienced a postoperative neurocognitive disorder during the postoperative period. Demographic and anesthesiologic variables are reported in the Table.

**Table.** Demographic and Anesthesiologic Variables of the 14 Patients Included in the Study

Age (y)	59.5 [55.3–65.2]
Weight (kg)	67.0 [48.6–81.2]
Height (cm)	165 [158–172]
BMI (kg/m^2^)	25.4 [19.6–30.8]
ASA physical status (I/II)	14/0
CeP stable (μg.mL^−1^)	2.75 [1.92–3.48]
CeR stable (ng.mL^−1^)	3 [3–3]
BIS	
baseline	35.6 [34.7–43.5]
CeK =1 µg.mL^−1^	46.4 [39.2–52.9]
maximum value	71.5 [68.7–75.4]
SEF_95_	
baseline	16.7 [15.8–17.3]
CeK =1 µg.mL^−1^	17.5 [16.6–18.0] Hz
maximum value	22.9 [21.9–23.6]
SPI	
baseline	41 [26–55]
CeK =1 µg.mL^−1^	39 [22–45]
Total propofol (mg)	939 [621–1246]
Total propofol (mg.kg^−1^)	14.2 [12.0–16.1]
Total remifentanil (mg)	1.6 [1.0–2.2]
Total remifentanil (mg.kg^−1^)	0.02 [0.02–0.03]
Total ketamine (mg)	38.5 [25.8–50.6]
Total ketamine (mg.kg^−1^)	0.57 [0.56–0.57]
Surgery time (min)	98.5 [59.2–132.8]

Data are reported as median [IQR].

Abbreviations: ASA, American Society of Anesthesiologists classification; BIS, bispectral index; BMI, body mass index; CeK, concentration at the effector site of ketamine; CeP, concentration at the effector site of propofol; CeR, concentration at the effector site of remifentanil; SEF_95_, spectral edge frequency.

**Figure 1.**
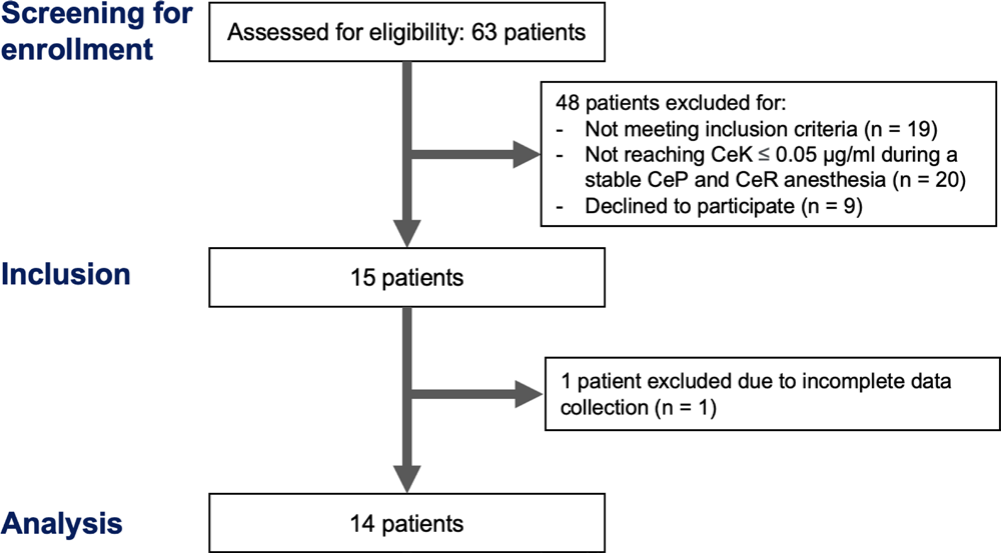
STROBE flow diagram. CeK indicates concentration at the effect-site of ketamine; CeP, concentration at the effect-site of Propofol; CeR, concentration at the effect-site of remifentanil; STROBE, Strengthening the Reporting of Observational Studies in Epidemiology.

### Density Spectral Array

Visual inspection of the DSA for all 14 patients showed a characteristic increase in alpha-band oscillatory activity (8–14 Hz) after propofol-induced anesthesia. After the administration of a ketamine bolus, the alpha oscillatory activity gave way to higher low-beta oscillatory activity (12–15 Hz). As CeK decayed, so did the low-beta oscillatory activity. After CeK returned to values <0.04 μg.mL^−1,^ the DSA showed a profile similar to the 1 before ketamine infusion, with high alpha and delta oscillatory activity. An exemplary DSA from a randomly chosen patient is shown in Figure 1, which also presents the corresponding CeK, BIS, and SEF_95_ trends.

### BIS and SEF_95_ Analysis and Correlation with CeK

We independently conducted the BIS and SEF95 analysis for the left and right electrodes. The results obtained were similar and we found no significant difference. Hence, we are reporting the results for the left temple electrode only.

**Figure 2.**
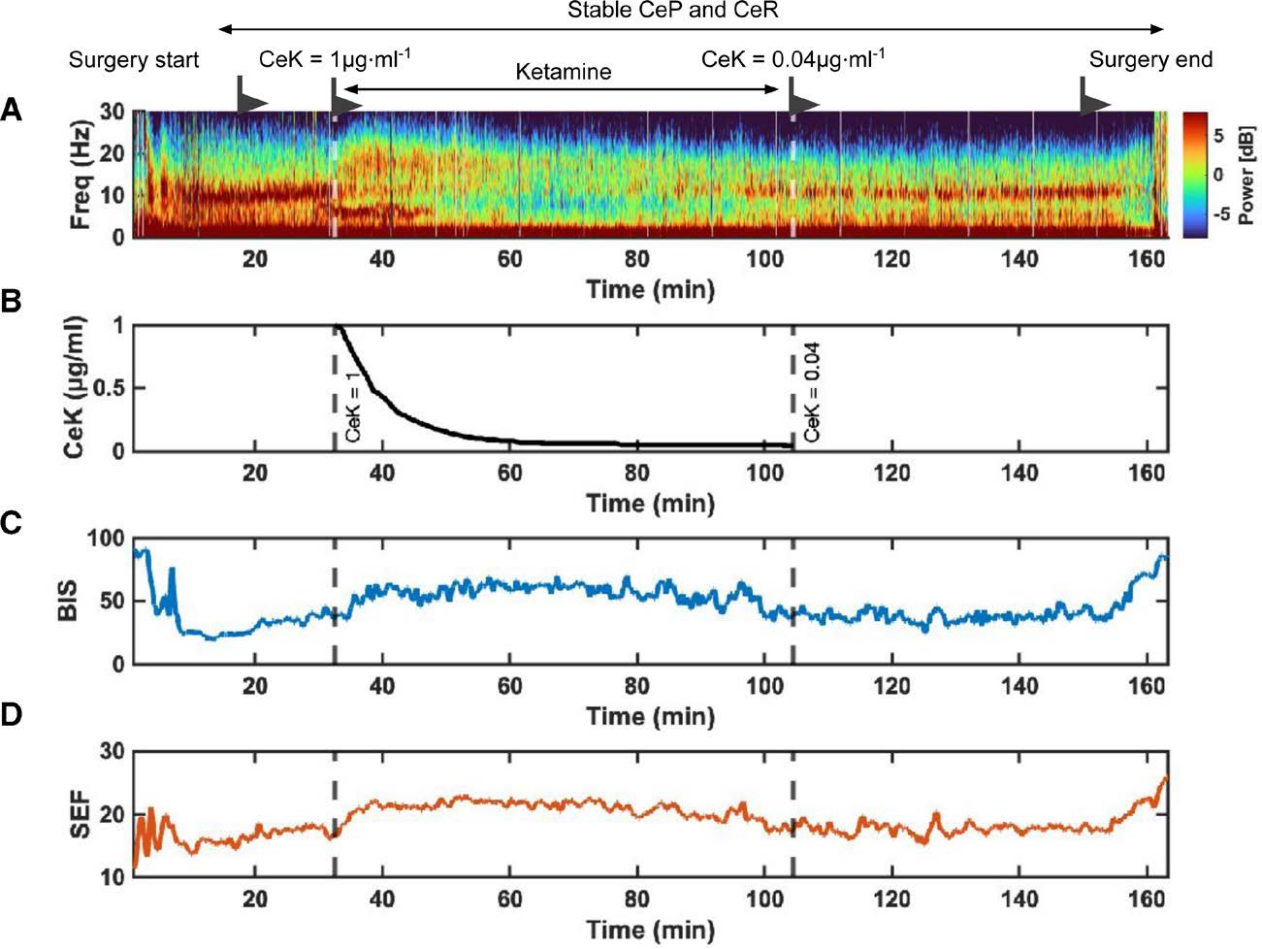
Exemplary DSA and trends of the CeK and processed EEG indices of 1 randomly chosen subject. The time interval shown includes the induction of general anesthesia, its maintenance, administering the ketamine bolus, and the end of surgery. The EEG information displayed corresponds to that recorded by the electrode placed on the left temple. A, DSA with the relevant events and the interval of observation marked. B, CeK as a function of time. C, BIS. D, SEF95. BIS indicates bispectral index; CeK, concentration at the effect-site of ketamine; DSA, density spectral array; EEG, electroencephalogram; SEF_95_, spectral edge frequency.

**Figure 3.**
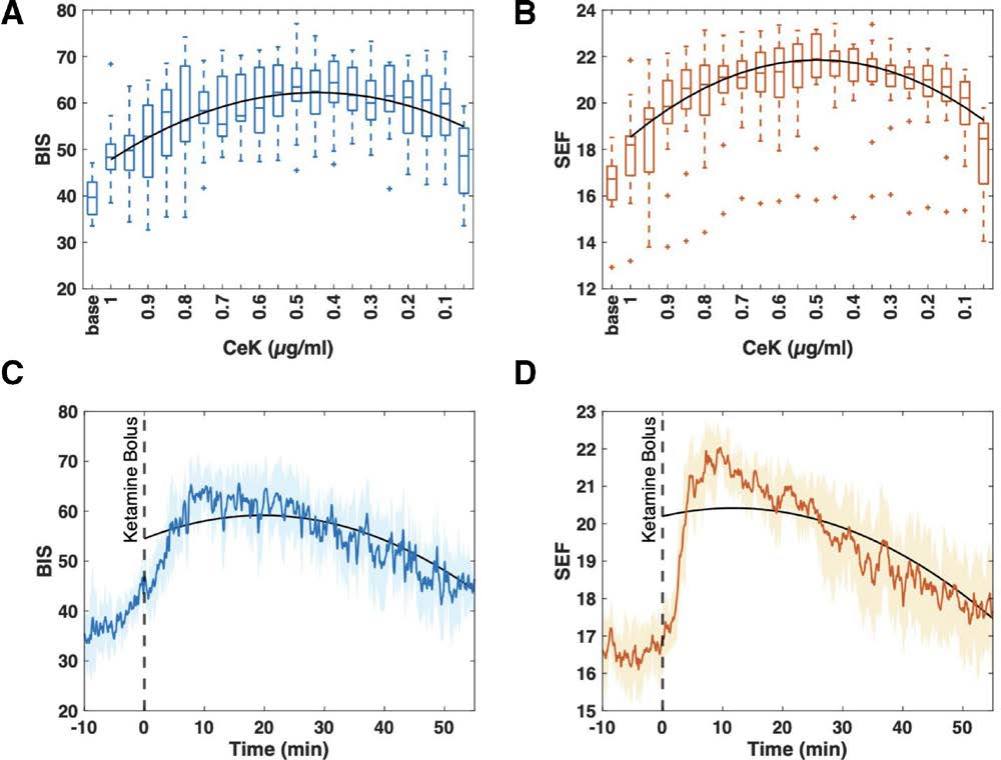
Trends of the processed EEG parameters BIS and SEF_95_ as a function of the CeK (A and B) and time (C and D). A, Change in BIS at the modeled CeK. The course can be fitted with a polynomial model of order 2: BIS = −47.0 (1−CeK) 2 + 52.1 (1−CeK) + 47.8 (R^2^ = 0.75). (coefficient [95% CI], −47.0 [−63.2 to −30.7], 52.1 [36.1–68.1], 47.8 [44.5–51.1]). B, Change in SEF_95_ at the modeled CeK. The course can be fitted with a polynomial model of order 2: SEF_95_ = −13.1 (1−CeK) 2 + 13.2 (1−CeK) + 18.5 (R^2^ = 0.90). (coefficient [95% CI], −13.1 [−15.1 to −11.1], 13.3 [11.2–15.2], 18.5 [18.1–18.9]). C, Change in BIS over the interval 10 min before until 55 min after ketamine delivery. The median time to reach the maximum BIS value is labeled tmax. D, Change in SEF_95_ over the interval 10 min before until 55 min after ketamine delivery. The median time to reach the maximum SEF_95_ value is labeled *t*_max_. The thick lines in (C) and (D) represent the group median, and the shaded area is the median absolute deviation. The black horizontal lines marked with an asterisk (*) in all subfigures indicate a significant change (*P* < .05) in the index compared to its baseline value before the ketamine infusion. BIS indicates bispectral index; CeK, concentration at the effect-site of ketamine; EEG, electroencephalogram; SEF_95_, spectral edge frequency.

A CeK of 1 µg.ml^-1^ was reached after a median [IQR] time of 2 minutes [1–2 minutes] after the administration of the ketamine TCI bolus, given at a median of 15.3 minutes [6.8–20.1 minute] after the start of surgery. The subsequent BIS and SEF_95_ behavior as a function of CeK showed characteristics of a biphasic trend (Figure 2A, 2B): Initially, the BIS significantly increased from a value of 46 [39.2–52.9] at CeK = 1 µg.ml^-1^ to a maximum of 71.5 [68.7–75.4] at a CeK = 0.22 µg.ml^-1^ [0.12–0.41 µg.ml^-1^] (*P* < .001). Similarly, the SEF_95_ significantly increased from a value of 17.5 Hz [16.6–18.0 Hz] at CeK = 1µg.ml^-1^ to a maximum value of 22.9 Hz [21.9–23.6 Hz] at a CeK = 0.43 µg.ml^-1^ [0.25–0.50 µg.ml^-1^]. The maximum BIS was reached 16.0 minute [10.2–22.7 minutes], and the maximum SEF 10.0 minute [8.62–14.1 minute] after CeK=1 µg.ml^-1^ (Table and Figure 3). After reaching their peak values, the BIS and SEF_95_ significantly dropped with decreasing CeK, reaching 48.6 [40.6–54.6] and 18.5 Hz [16.5–19.1 Hz], respectively, by the end of the observation period at a CeK = 0.05 µg.ml^-1^. The BIS’s final reported value was 12.6 [0.6–18.9] above its preketamine baseline value. Similarly, for the SEF_95,_ it lay 1.6 Hz [0.8–2.4 Hz] above its baseline. The biphasic trends of BIS and SEF_95_ matched on CeK showed a high coefficient of correlation when fitted with a polynomial model of order 2: BIS = –47.0 (1–CeK) ^2 + 52^.1 (1–CeK) + 47.8 (R^2^ = 0.75); SEF_95_ = –13.1 (1–CeK) ^2 + 13^.2(1–CeK) + 18.5 (R^2^ = 0.90).

**Figure 4.**
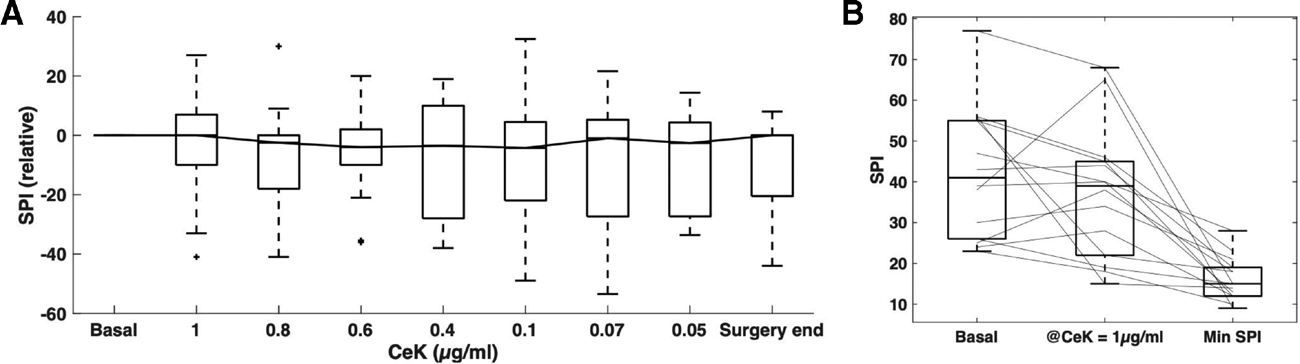
SPI as a function of ketamine dose at the modeled CeK. There were no significant ketamine-related variations in the SPI. CeK indicates concentration at the effect-site of ketamine; SPI, surgical pleth index.

Preceding ketamine administration, the target BIS value was set to 40 to 60 since a BIS within this range has been regarded as adequate during general anesthesia for adult patients.^15^ Ketamine administration was associated with an increase in the BIS beyond 60. The median [IQR] time interval from the administration of ketamine to the recording of a BIS value greater than 60 was 4.4 minutes [3.0–5.5 minutes]. A BIS >60 was first reached at a CeK of 0.93 μg.mL^−1^ [0.82–0.97 μg.mL^−1^]. The BIS was observed to remain above the 60 threshold for several minutes to subsequently drop (BIS <60) at a CeK of 0.06 μg.mL^−1^ [0.05–0.06 μg.mL^−1^], 44.6 minutes [36.5–57.7 minutes] after the administration of the ketamine bolus (Figure 2C, 2D).

### SPI Analysis and Correlation with CeK

Figure 4A shows the raw SPI trends recorded at a per-minute resolution. Figure 4B shows the SPI as a function of CeK in the range CeK = 1 μg.mL^−1^ to CeK = 0.04 μg.mL^−1^ in decreasing intervals of 0. 2 ± 0.1 μg.mL^−1^. The median SPI shows no statistically significant change from baseline and no biphasic trend (Figure 4A). We found no significant change between the basal SPI and the SPI at the time when CeK = 1 μg.mL^−1^.

## DISCUSSION

### Summary of the Results

Our analysis revealed that administering a ketamine bolus significantly increased BIS and SEF. In contrast, SPI was not observed to change significantly from its preketamine baseline value.

### Changes in BIS and SEF_95_

The effect of a ketamine dose on brain activity during propofol anesthesia has been previously described. Several studies report an increase in the BIS after 3 to 8 minutes,^7,20^ 15 to 20 minutes,^5^ and 30 minutes^17^ after ketamine infusions in the range 0.4 to 1 mg.kg^−1^. The SEF_95_ has also been observed to increase after ketamine administration.^21^ This agrees with our findings, which showed a significant increase in the BIS and SEF_95_.

In this study, the administration of a single ketamine bolus was associated with a biphasic trend for the BIS and SEF_95_, suggesting a dose-dependent effect of ketamine on the brain’s cortical activity. There are no previous reports of a dose-response analysis of a single dose of ketamine on EEG-derived indices evaluated as a function of the CeK. Our results revealed that while BIS and SEF_95_ exhibited no significant changes from baseline at the target and maximum CeK of 1.0 µg.ml^-1^, significantly higher values were reached after a few minutes, with the peak median value occurring at a CeK of 0.22 and 0.43 µg.ml^-1^ respectively. After reaching their maximum value, the processed EEG indices decreased with decreasing CeK. The second-order polynomial models’ high correlation coefficients (R2≥0.75) indicate that the dose-response trend for BIS and SEF_95_ showed a strong nonlinear relationship between the CeK and the index. At the lowest CeK recorded (0.04–0.05 µg.ml^-1^), BIS and SEF_95_ were significantly higher than their baseline value before ketamine administration. However, since BIS and SEF_95_ have a downward trend with CeK in the parabolic descending part, it would potentially have returned to baseline values. Behavior is consistent with the changes observed in the DSA. After the administration of ketamine, the DSAs showed that the slow-delta and alpha oscillations, a characteristic EEG pattern for propofol-anesthesia,^4^ switched to a pattern with dominant oscillatory activity in the beta to gamma range that faded with ketamine clearance and finally returned to the baseline condition.

The observed biphasic dose-response for BIS and SEF_95_ may be explained by ketamine’s different effects on cortical brain activity. At low doses, ketamine preferentially inhibits NMDA-mediated glutamatergic inputs to gamma-aminobutyric acidergic inhibitory interneurons, leading to aberrant excitatory activity in the cortex, hippocampus, and limbic system.^2,4^ This is manifested in the EEG as increased gamma oscillations.^2,4^ At higher doses, ketamine also blocks NMDA receptors on excitatory pyramidal neurons found in various locations in the central nervous system, in particular, the glutamatergic projections from the parabrachial nucleus and the medial pontine reticular formation in the brainstem to the thalamus and to the basal forebrain.^16,22,23^ These are potent, excitatory arousal pathways,^2,4^, and their inhibitions are revealed by profound slow-delta oscillations alternating with gamma oscillations—the so-called “gamma burst” EEG pattern.^24^ A “gamma burst” EEG pattern alone,^5^ or associated with increased theta oscillations and decreased alpha/beta oscillations,^25^ was observed after ketamine administration. After ketamine administration, the EEG pattern of ketamine overlaps that of propofol, characterized by slow delta and alpha oscillations.^2,4^ The slow delta oscillations most likely represent hyperpolarization of the thalamus and cortex due to direct inhibition of pyramidal neurons by the anesthetics in the cortex, thalamus, and brainstem.^22^ The alpha oscillations probably represent activity between the thalamus and the cortex.^26^ Unconsciousness is maintained by impairing neuronal communication within and between brain regions.^2,4^ Consequently, BIS and SEF_95_ changed according to the CeK. They were lower at the initially high concentration and increased with falling concentrations. The delay effect, with different timing in changes of EEG indexes, may also be due to differences between BIS and SEF_95_. SEF_95_ is a less processed parameter than BIS and can be directly derived from the power spectrum. Furthermore, some studies described a time delay of BIS calculation, attesting an average delay of 30 seconds,^27,28^ but the intrinsic algorithms for BIS and SEF_95_ generation remain unknown. This suggests that SEF_95_ may be a more reliable parameter to indicate short-term changes in cortical oscillatory activity.

### Time Delay in the Maximum BIS and SEF95: Clinical Implication

The observed time delay between administering an analgesic ketamine bolus of median dose 0.57 mg.kg^−1^ and the peak BIS and SEF_95_ value has important clinical implications. The BIS increased above the 60-point threshold 4.4 minutes [3.0–5.5 minutes] after the administration of the ketamine bolus at a CeK of 0.93 µg.ml^-1^ [0.82–0.97 µg.ml^-1^], with the peak value occurring at time 16.0 minute [10.3–22.7 minutes] after the bolus. Subsequently, the median BIS value dropped below the 60-point threshold 44.6 minutes [36.5–57.7 minutes] after ketamine administration, at a CeK of 0.06 µg.ml^-1^ [0.05–0.06 µg.ml^-1^]. Similarly, SEF reached its maximum value 10.0 minute [8.6–14.1 minute] after ketamine administration. Thus, anesthesiologists should be aware that after administering an analgesic ketamine bolus, BIS values can rise above the BIS = 60 threshold for several minutes. However, these values may not reflect a lighter hypnotic level. Hence, variations in the anesthetic plan during this time interval as a response to an increasing BIS value should be carefully evaluated. A misinterpretation of higher EEG-derived parameters can encourage anesthesiologists to increase the propofol infusion rate being administered, which, in high doses, may lead to a higher risk of burst suppression episodes. These have been linked to a greater incidence of postoperative delirium.^29–31^

### Changes in SPI

No significant changes were observed for SPI after ketamine administration during stable TIVA-TCI, although there was a decrease shortly after the CeK peak (Figure 4).

Our study directly addresses the effect of CeK on SPI, an index used to assess stress response and nociception during surgery. Remifentanil was infused during the induction and maintenance phases. It acts on opioid receptors in the brain and spinal cord, enhancing descending inhibition of nociceptive inputs beginning at the level of the periaqueductal gray and blocking afferent nociceptive inputs into the spinal cord.^2^ In addition, ketamine also acts on the spinal cord, blocking NMDA-mediated glutamatergic nociceptive signals from peripheral afferent neurons in the dorsal root ganglion to projecting neurons.^2^ Thus, both drugs concurred to limit the effect of stress response and nociception-related surgical stimulation on SPI. This may explain why the SPI remained relatively stable and showed no clear indication of a downward trend after administering a ketamine bolus. The fact that SPI did not decrease after ketamine bolus probably reflects the fact that remifentanil TCI was already sufficient to guarantee adequate analgesia for the surgical stimulation, and analgesic properties of ketamine were insufficient to provide a decrease in our in-range values of SPI reached by remifentanil TCI. However, since we did not consider heart rate or mean blood pressure, we cannot exclude that an increase in sympathetic activity described with ketamine^32^ can underestimate the analgesic effect of ketamine on SPI.

### Limitations

This study presents some limitations. Firstly, it is an observational study, and the lack of a control group, with patients only being administered a placebo during TIVA, may have limited the correct interpretation of our findings. However, the ketamine effects on the EEG being discussed have been observed independently in studies that did^5–7,21^ or did not^20^ include a control group. Secondly, we considered the estimated CeK without blood sampling of plasma concentrations of ketamine. Some have regarded the performance of the Domino TCI model during controlled low-dose ketamine infusions as suboptimal compared with that of other models,^10^ and the association with propofol and remifentanil can have exacerbated its performance on CeK calculation. This study used the Domino model since it is the only model approved for clinical practice at the hospital where the study was conducted. However, while this may have incurred some inaccuracies in the calculation of the CeK, it is unlikely they would have played a decisive role in the results. Thirdly, an age-group analysis was not performed. EEG indices are believed to show higher values in older patients,^33,34^ and their response to a changing drug concentration may be affected by aging. In addition, ketamine-induced changes in the EEG have been observed to be more prominent in elderly patients who were not cognitively impaired at baseline than those who were.^35^ However, the subjects in this study were of a median age of 59.5 years [55.3–65.2 years], with only 2 patients older than 65 years. Thus, variations due to age are not thought to play a decisive role. Furthermore, surgical stimuli with augmented nociception levels might have increased BIS, SEF_95,_ and SPI. However, the ketamine bolus was delivered 15.3 minutes [6.8–20.1 minute] after surgery started. This was after the first surgical demolition phase of mastectomy/quadrantectomy. Breast surgery is not a highly invasive surgery and does not trigger a strong stress response as a reaction to pain.^36,37^ Hence, we know that nociception events could have selectively influenced our observations but not the overall result.

The sample size of 14 patients was derived for the primary hypothesis that ketamine increases the BIS. For the assessment of the indices over the entire concentration range, we did not estimate the sample size. So, we could not report significant results, for example, increased indices at the very low ketamine concentrations, because of insufficient effect sizes. A more detailed index-to-concentration correlation needs to be performed in the future. The correction for multiple comparisons was based on the “neighbor” approach and not on the classical post hoc testing to prevent type I errors. We followed the suggestion of not performing the post hoc testing when the expense of false negatives seems higher than reporting false positives.^19^ We did not consider covariates or construct mixed effect models because of the small sample size, as the model’s complexity would increase. These analyses need to be performed in a larger cohort.

Another limitation is that this study considered only female patients. Although preclinical studies may have identified sex differences in response to ketamine, the data situation is not clear.^38^ Recent trials could not confirm gender differences.^39,40^ Future studies should analyze sex differences in the power spectrum after a ketamine bolus during propofol-remifentanil TIVA-TCI. Although after the strict protocol described in the methods section, the processed BIS and SPI indices distill complex information into a single number. This can generally lead to similar indices (or index combinations) for different clinical situations. Based on our strict protocol, we are confident to largely have had control over it.

## CONCLUSIONS

This study showed that administering an analgesic ketamine bolus during propofol-remifentanil-general anesthesia is associated with significant changes in the BIS and SEF_95_. Their peak values were not observed at the target CeK of 1 µg.ml^-1^ but at lower CeKs in the 0.2 to 0.5 µg.ml^-1^ range, declining shortly after that, after a parabolic trend.

## ACKNOWLEDGMENTS

The authors would like to thank Michele Ruol, MD, and Sara De Piccoli, MD, for their efforts in data collecting.

## DISCLOSURES

**Conflicts of Interest:** None. **Funding:** None. **This manuscript was handled by:** Jiro Kurata, MD, PhD.

## Supplementary Material
